# Sex discrepancy in the reduction of mucosal‐associated invariant T cells caused by obesity

**DOI:** 10.1002/iid3.393

**Published:** 2020-12-17

**Authors:** Jianyun Liu, Hongmei Nan, Randy R Brutkiewicz, Jose Casasnovas, Kok Lim Kua

**Affiliations:** ^1^ Department of Microbiology and Immunology Indiana University School of Medicine Indianapolis Indiana USA; ^2^ Department of Global Health, Richard M. Fairbanks School of Public Health Indiana University Indianapolis Indiana USA; ^3^ Indiana University Melvin and Bren Simon Cancer Center Indiana University Indianapolis Indiana USA; ^4^ Department of Pediatrics Indiana University School of Medicine Indianapolis Indiana USA

**Keywords:** MAIT, obesity, sex discrepancy

## Abstract

**Introduction:**

Gut microbiota has been reported to contribute to obesity and the pathology of obesity‐related diseases but the underlying mechanisms are largely unknown. Mucosal‐associated invariant T (MAIT) cells are a unique subpopulation of T cells characterized by the expression of a semi‐invariant T cell receptor (TCR) α chain (Vα19 in mice; Vα7.2 in humans). The expansion and maturation of MAIT cells require the gut microbiota and antigen‐presenting molecule MR1, suggesting that MAIT cells may play a unique role in bridging gut microbiota, obesity, and obesity‐associated inflammation.

**Methods:**

The levels of human MAIT cells from obese patients, as well as mouse MAIT cells from obese mouse models, were determined by flow cytometry. By comparing to controls, we analyzed the change of MAIT cells in obese subjects.

**Results:**

We found obese patients had fewer circulating MAIT cells than healthy‐weight donors and the difference was more distinct in male patients. Consistently, male mice (but not female mice) have shown reduced MAIT cells in the liver and adipose tissue after a 10‐week Western diet compared to mice on a control diet. We also explored the possibility of utilizing high‐throughput technology (i.e., quantitative polymerase chain reaction [qPCR]), other than flow cytometry, to determine the expression levels of the invariant TCR of human MAIT cells. But a minimal correlation (*R* ^2^ = 0.23, *p* = .11) was observed between qPCR and flow cytometry data.

**Conclusion:**

Our study suggests that there is a sex discrepancy in the impact of obesity on MAIT cells: MAIT cells in male (but not female) humans and male mice are reduced by obesity.

## Graphical abstract

It has been previously reported that MAIT cells are reduced in obese patients. We also found fewer MAIT cells in both preclinical obese mouse models and obese patients. Moreover, our data suggest there is a sex discrepancy that the reduction of MAIT cells caused by obesity is only observed in males but not females in both preclinical mouse models and human subjects.

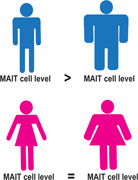

**Abbreviations**
BMI: body mass index
DIO: diet‐induced obesity
MAIT: mucosal‐associated invariant T cells
MNC: mononuclear cells
NK: natural killer
NKT: natural killer T
PBMC: peripheral blood mononuclear cell
PCR: polymerase chain reaction
TCR: T cell receptor
VAT: visceral adipose tissue

## 1 INTRODUCTION

The global incidence of obesity has been steadily increasing for the past 10 years.^1^, ^2^ According to WHO, over 650 million adults aged 18 years and older, representing 13% of adults worldwide, were obese in 2016.^3^ Once considered a health problem only in developed countries, obesity has spread to developing countries. In 2019, 38 million children under the age of 5 years were overweight or obese with nearly half of them living in Asia.^3^ Obesity clearly will continue to be an important worldwide health issue for years to come. Importantly, obesity increases the risks of diseases such as coronary artery diseases, hypertension, stroke, type 2 diabetes, and certain types of cancer, including that of the pancreas, colon and rectum, and breast.^4^ Obesity is also a risk factor for critical illness and hospitalization upon infection with H1N1 influenza as well as coronavirus disease 2019 (COVID‐19).^5^, ^6^ Obesity is accompanied by a chronic proinflammatory state that would be a potential mechanism that explains the increased risk of cancer and complications during viral infections.^7^, ^8^ The immune response in obese people is also altered,^9^, ^10^ which may suppress antitumor and viral immunity. Nonetheless, the mechanisms linking obesity to inflammation and immunosuppression remain unclear.

Mucosal‐associated invariant T (MAIT) cells are a subpopulation of lymphocytes that are abundant in the mucosal tissues.^11^ MAIT cells are evolutionarily conserved innate‐like T cells expressing a semi‐invariant T cell receptor (TCR) α chain (Vα19 in mice and Vα7.2 in humans). The antigen‐presenting major histocompatibility complex class I‐related molecule MR1 is required for MAIT cell development.^11^ It has also been demonstrated that microbial vitamin B metabolites are the MR1‐presented antigens for MAIT cell activation.^12^ Upon activation by bacteria‐infected antigen‐presenting cells, MAIT cells rapidly secrete proinflammatory cytokines (e.g., interferon‐γ, tumor necrosis factor‐α, and Granzymes A and B).^11^ The expansion and maturation of MAIT cells requires commensal bacteria, as these cells are scarce in germ‐free animals.^11^ Few MAIT cells (~0.03%) are found in human cord blood, and these MAIT cells display a naïve phenotype.^13^, ^14^ However, significantly more MAIT cells (>1.3%) with a memory phenotype are present in adult blood.^13^, ^14^ Reduced MAIT cell levels have been reported in microbial infections and in autoimmune diseases,^15^, ^16^, ^17^, ^18^, ^19^ suggesting MAIT cells may play critical roles in these diseases. Two recent reports also demonstrate profound MAIT cell abnormalities in obese patients.^20^, ^21^ However, the impact of sex‐difference on MAIT cells in response to obesity has not been studied.

In the present study, we utilized a Western diet‐induced obesity (DIO) mouse model to determine how MAIT cells in mice were impacted by increased adiposity. Moreover, using flow cytometry, we compared the frequencies of MAIT cells in the peripheral blood between obese patients and healthy donors. We also explored the possibility of utilizing quantitative polymerase chain reaction (qPCR) to measure levels of Vα7.2 TCR of MAIT cells in human blood, because qPCR is the gold standard of gene expression quantification and also compatible with high‐throughput methods.^22^

## 2 METHODS

### 2.1 Animals and diet‐induced obesity model

All animal procedures were approved by the Indiana University School of Medicine Institutional Animal Care and Use Committee and conformed to the regulations of the Animal Welfare Act and the National Institutes of Health Guide for the Care and Use of Laboratory Animals. Five‐week old male and female C57BL/6 mice were purchased from The Jackson Laboratory and housed in a pathogen‐free, temperature controlled, 12‐h light–dark cycled animal care facility with free access to water and food. Animals were randomized to receive standard chow (2018SX; Envigo) as a control, or a Western diet (42% fat calorie, TD.88137; Envigo) for 10 weeks to induce obesity. Animals were euthanized via CO_2_ inhalation and tissues (liver and visceral adipose) were collected for experimental analyses.

### 2.2 Blood samples

This study was approved by the Institutional Review Board of Indiana University. Blood samples from obese patients with body mass index (BMI) greater than 30 (OB group) and healthy donors (HD group) matched by age, sex, race/ethnicity, and fasting status (≥8 h) were collected by the Indiana Biobank (Table 1). To avoid the possibility of other factors influencing the immune system, we excluded patients with cancer, chronic infections (e.g., human immunodefeciency virus and hepatitis B virus), type 2 diabetes, history of using tobacco products, or on immunosuppressive drugs.

**Table 1:** Characteristics of the study cohort

**Sex**	**Group**			
	**Obese patients (BMI > 30)**		**Healthy donors (BMI < 26)**	
	**Number**	**Age**	**Number**	**Age**
Female	14	26–71 years (mean, 48)	14	19–74 years (mean, 46)
Male	8	31–70 years (mean, 51)	5	21–73 years (mean, 42)

Abbreviation: BMI, body mass index

### 2.3 Antibodies

PE/Cy5‐conjuated anti‐human CD3 and a human NKT cell‐specific antibody (6B11) were purchased from BD Biosciences. PE‐conjugated anti‐human Vα7.2 TCR (Clone 3C10) and AlexFluo 488‐labeled anti‐human CD161 (Clone HP‐3G10) were obtained from Biolegend. PE‐conjugated anti‐human Vα24 TCR and fluorescein isothiocyanate (FITC)‐conjugated anti‐human Vβ11 TCR were purchased from ImmunoTech. Mouse‐specific FITC‐anti‐B220 (Clone RA3‐6B2), FITC‐anti‐F4/80, PE‐anti‐TCRβ (Clone H57‐597), and Pacific Blue‐anti‐CD44 (Clone IM7) were from Biolegend.

### 2.4 MAIT and NKT cell culture

MAIT cell cultures were performed as previously described.^23^ Briefly, MAIT cells (CD161^+^Vα7.2^+^) from human peripheral blood mononuclear cells (PBMCs) were sorted by flow cytometry and cocultured with irradiated PBMCs in the presence of fixed *E. coli* and recombinant human interleukin‐2 (IL‐2) for 3 weeks. A natural killer T (NKT) cell line was also expanded from PBMCs as previously described.^24^ In brief, NKT cells were first isolated from PBMCs by flow cytometry using a human NKT cell‐specific antibody (6B11). The cells were stimulated with irradiated allogeneic human PBMCs in the presence of α‐glactosylceramide (Alexis Biochemicals) and recombinant human IL‐2 and cultured for at least 3 weeks.

### 2.5 Mononuclear cell isolation from mouse liver and adipose tissue

Mononuclear cells (MNCs) were isolated from mouse liver as previously described.^25^ Briefly, after perfusion with PBS, liver tissue was harvested, homogenized and pressed through a 70 µm cell strainer. Liver MNCs were then isolated from the homogenates by gradient centrifugation using 37.5% percoll (GE Healthcare). Adipose MNCs were prepared following a previously published protocol.^26^ Visceral adipose tissue was harvested, weighed and then minced. After collagenase digestion, the homogenates were passed through a 100 µm cell strainer. Adipose MNCs were pelleted by centrifugation.

### 2.6 Flow cytometry

PBMCs were isolated from each blood sample using Ficoll‐hypaque gradient centrifugation. To identify MAIT cells, human PBMCs from both obese patients and healthy donors were stained with PE/Cy5‐conjuated anti‐CD3, PE‐conjugated anti‐Vα7.2, and AlexFluo 488‐conjugated anti‐CD161 mAbs at 4°C for 30 min. To stain for NKT cells, cells were incubated with PE‐conjugated anti‐TCR Vα24 and FITC‐conjugated anti‐TCR Vβ11 for 30 min at 4°C. Mouse MNCs were prepared from mice on a Western or control diet as described in the previous section. The cells were then stained with FITC‐anti‐B220, FITC‐anti‐F4/80, PE‐anti‐TCRβ, Pacific Blue‐anti‐CD44, and APC‐conjugated MR1 tetramers (NIH Tetramer Core Facility) at 4°C for 30 min. All samples were acquired on an LSR4 flow cytometer (BD Biosciences) and analyzed by using FlowJo 10 software (Tree Star).

### 2.7 RNA extraction

CD3^+^ T cells from human PBMCs were isolated using magnetic bead‐associated sorting (Miltenyi Biotec). Total RNA was extracted from these T cells using the RNeasy kit (Qiagen). RNA from these samples was used as template for the synthesis of cDNA using the Transcriptor First Strand cDNA Synthesis Kit (Roche).

### 2.8 Quantitative polymerase chain reaction

Gene‐specific primers and probe for glyceraldehyde 3‐phosphate dehydrogenase (GAPDH) were obtained from Thermo Fisher Scientific. The primers and TaqMan probe sets for MAIT and NKT cells (Table 2) were designed based on previous publications^27^, ^28^ and custom‐ordered from Thermo Fisher Scientific. The Taqman PCR master mix (Thermo Fisher Scientific), together with primers and TaqMan probes, were added to each sample for the PCR reaction and run on the QuantStudio 6 Flex Real‐Time PCR System (Thermo Fisher Scientific). Upon completion of the PCR reactions, the value of each gene of interest was calculated as $2^{∆C_{t}(\mathrm{GAPDH})}$. All samples were analyzed in duplicate.

**Table 2:** Primers and probes for human MAIT and NKT cells

**Name**	**Forward primer**	**Reverse primer**	**Probe**
TRAV 1.2 (MAIT)	TCCTTAGTCGGTCTAAAGGGTACA	GGCAGACAGACTTGTCACTGGAT	GATAGCAACTATCAGTTAATC
TRAV 10 (NKT)	GATATACAGCAACTCTGGATGCA	GGCAGACAGACTTGTCACTGGAT	TGGGGAGGCTATACTTTGGA

### 2.9 Statistical methods

All statistical analyses were performed using GraphPad Prism 8. Groups were compared using the Mann–Whitney unpaired *t* test. Data are shown as means ± standard deviation. Parameter correlation was determined using Pearson's correlation coefficient. *p* < .05 were considered statistically significant.

## 3 RESULTS

### 3.1 Reduced circulating MAIT cells in obese patients

**Figure 1:**
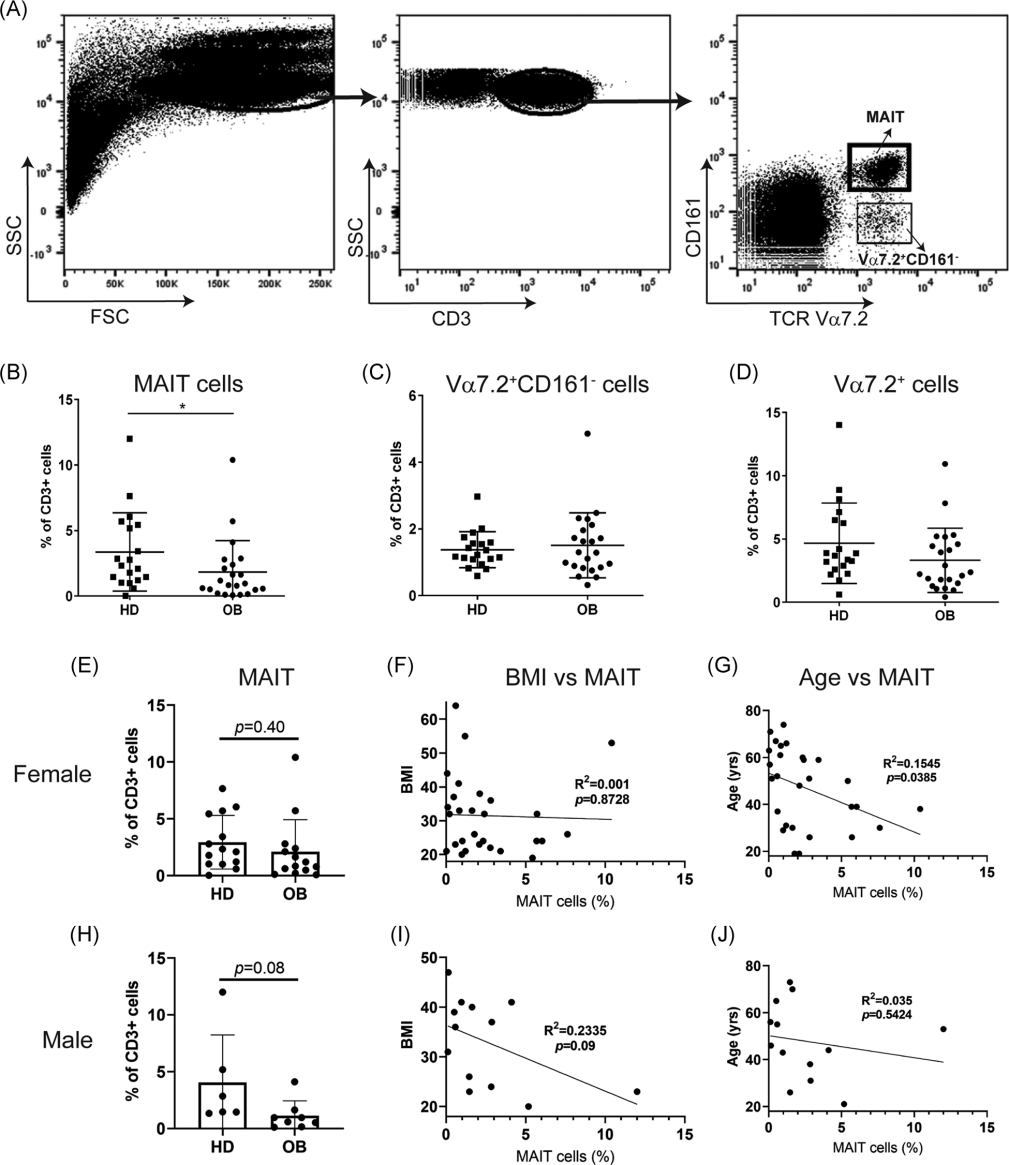
Reduced circulating MAIT cells in human obese subjects. (A) Peripheral blood mononuclear cells (PBMCs) were isolated and stained with CD3‐, CD161‐, and TCR Vα7.2‐specific mAbs and analyzed by flow cytometry. Frequencies of Vα7.2^+^CD161^+^MAIT cells (B), Vα7.2^+^CD161^−^ cells (C), and Vα7.2^+^ cells (D) from both healthy donors (HD) and obese patients (OB) are summarized. Frequencies of MAIT cells from female (E) and male (H) subjects are shown. Correlation of the frequencies of MAIT cells to BMI in female (F) and male (I) subjects, or to age in female (G) and male (J) subjects, are shown. The data are shown as the mean ± *SD*. Each dot represents an individual. *p < .05

Human blood samples were obtained from obese patients and healthy controls (Table 1). Consistent with previous reports,^20^, ^21^ we found significantly fewer MAIT cells (TCR Vα7.2^+^CD161^+^) in blood samples from obese patients compared to healthy donors (Figure 1A and 1B). On the other hand, the frequency of TCR Vα7.2^+^CD161^−^ cells in obese subjects and healthy controls was quite similar (Figure 1C). Total TCR Vα7.2^+^ cells from obese patients also differed modestly from healthy controls (Figure 1D). Interestingly, when the same data were analyzed based on sex, we found that MAIT cells in female obese subjects were not statistically different from female healthy controls (*p* = .40; Figure 1E), while MAIT cells from male obese subjects were substantially reduced comparing to male healthy subjects (*p* = .08; Figure 1H). MAIT cells from female subjects showed negative correlation with age (*R* ^2^ = 0.1545, *p* = .0385; Figure 1G) but no correlation with BMI (*R* ^2^ = 0.001, *p* = .8728; Figure 1F). On the contrary, MAIT cells from male subjects were inversely correlated with BMI (*R* ^2^ = 0.2335, *p* = .09; Figure 1I) but not with age (*R* ^2^ = 0.035, *p* = .5424; Figure 1J). Overall, these results suggest that obesity reduces the number of circulating MAIT cells and this appears to be more impactful in male obese patients.

### 3.2 qPCR analysis of MAIT cells is not adequately sensitive for distinguishing small numerical differences

**Figure 2:**
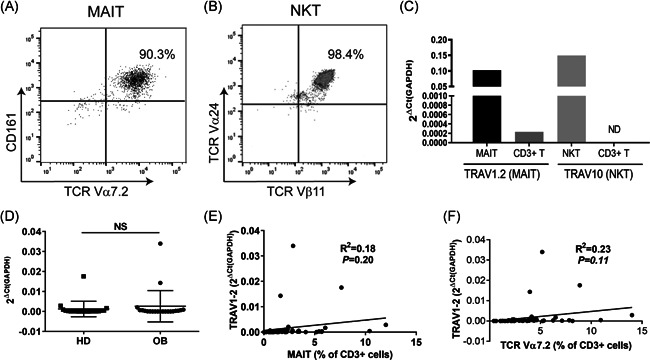
Comparable levels of *TRAV 1.2* messemger RNA (mRNA) expression by quantitative polymerase chain reaction (qPCR) in healthy and obese subjects. (A) MAIT cells (Vα7.2^+^CD161^+^) from human PBMCs were sorted by flow cytometry. These cells were cocultured with irradiated PBMCs in the presence of fixed *E. coli* and IL‐2 for 3 weeks. The enrichment of MAIT cells was confirmed by flow cytometry using CD161‐ and Vα7.2‐specific mAbs. (B) A human NKT cell line was expanded from PBMCs as described in the Materials and Methods. The purity of NKT cells was determined by flow cytometry using Vα24‐ and Vβ11‐specific mAbs. (C) Specific primers and probes were designed for qPCR analysis of *TRAV1.2* (for MAIT cells) and *TRAV10* (for NKT cells) mRNA expression. Enriched MAIT and NKT cells from (A) and (B), as well as CD3^+^ T cells, were analyzed by qPCR for the expression of *TRAV1.2* and *TRAV10*. ND: not detectable. (D) CD3^+^ T cells were enriched from blood samples of both HD and OB groups, using magnetic beads‐associated sorting, and analyzed by qPCR for *TRAV1.2*. The $2^{∆C_{t}(\mathrm{GAPDH})}$ values of all samples were summarized. (E) Correlation of the $2^{∆C_{t}(\mathrm{GAPDH})}$ values to MAIT cell frequencies in CD3^+^ T cells of all samples. (F) Correlation of the $2^{∆C_{t}(\mathrm{GAPDH})}$ values to TCR Vα7.2^+^ cell frequencies in CD3^+^ T cells of all samples. GAPDH, glyceraldehyde 3‐phosphate dehydrogenase; IL, interleukin; NS, not significant; PBMC, peripheral blood mononuclear cell

MAIT cells are normally identified by staining PBMCs with TCR‐specific antibodies and performing flow cytometry, as shown in Figure 1A. Real‐time PCR has been commonly used to quantify murine MAIT cells,^29^ but is used less frequently in humans. MAIT cells are very similar to another well‐studied subpopulation of T cells called NKT cells, which are also invariant T cells.^30^ It was previously reported that obesity also reduces the number of NKT cells.^28^, ^31^, ^32^, ^33^, ^34^ To determine whether qPCR can be used to quantify human MAIT cells in blood, we first designed specific primers and probes for MAIT and NKT cells based on previous publications displaying the DNA sequences of MAIT cell TCR (Vα7.2, *TRAV1.2*) and NKT cell TCR (Vα24, *TRAV10*),^27^, ^28^ as shown in Table 2. To validate these primers and probe sets, we obtained human MAIT (Figure 2A) and NKT (Figure 2B) cell lines that were greater than 90% pure. These two cell lines, together with CD3^+^ T cells isolated from healthy human blood, were analyzed by qPCR for the expression of *TRAV1.2* (MAIT) and *TRAV10* (NKT). As expected, the expression of *TRAV1.2* in bulk CD3^+^ T cells was very low, much higher in the MAIT cell line (Figure 2C), but undetectable in the NKT cell line (data not shown). On the other hand, *TRAV10* expression was high in NKT cell line, but undetectable in either the MAIT cell line (data not shown) or bulk CD3^+^ T cells (Figure 2C). We then measured MAIT cell levels in the obese cohort of this study using qPCR. CD3^+^ T cells were isolated from the same obese blood samples and healthy controls shown in Figure 1. These cells were used for total RNA isolation and qPCR. To our surprise, the expression of *TRAV1.2* in the obese and healthy groups was the same (Figure 2D). In addition, the frequencies of MAIT cells according to flow cytometry analysis were weakly correlated with *TRAV1.2* expression (*R* ^2^ = 0.18, *p* = .20; Figure 2E). *TRAV1.2* encodes the expression of TCR Vα7.2^27^; however, *TRAV1.2* expression only weakly correlated with Vα7.2^+^ cell frequencies (*R* ^2^ = 0.23, *p* = .11; Figure 2F). Our data suggest that a qPCR analysis of MAIT cells may be useful for distinguishing large differences, such as approximately 90% (MAIT cells) versus <5% (NKT and CD3^+^ T cells) data in Figure 2C, BUT this tool is not sufficiently sensitive to distinguish the small numerical differences identified by flow cytometry between obese and healthy control groups (4% vs. 2%).

### 3.3 Increased fat tissue in mice on a Western diet

**Figure 3:**
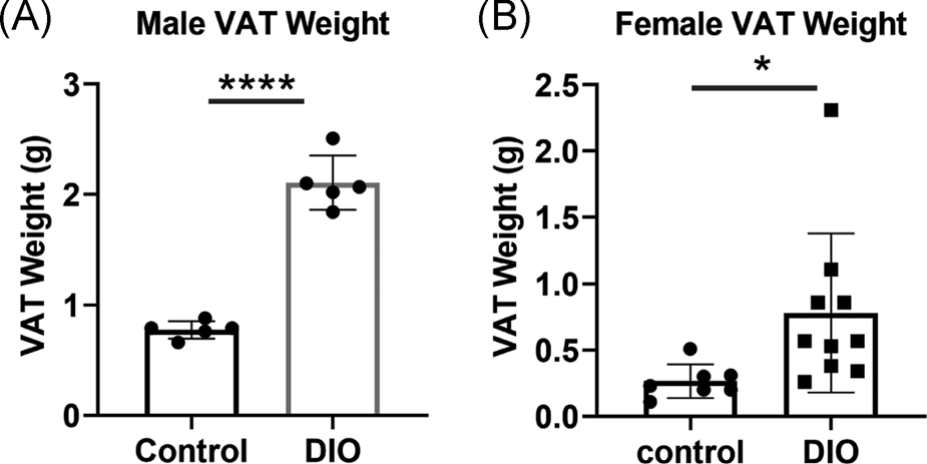
Increased adipose tissue in mice on a Western diet. Five‐week old male (A) and female (B) mice were on a Western diet to induce obesity (DIO) or control diet for 10 weeks. The weights of visceral adipose tissue (VAT) are shown as the mean ± standard deviation. Each dot represents an individual animal. *p < .05, ****p < .0001

To study the effect of obesity, we next evaluated MAIT cells in a preclinical model of DIO. Five‐week‐old male and female wild‐type C57BL/6 mice received a Western diet (DIO) or standard chow diet (control) for 10 weeks. Visceral adipose tissue (VAT) was collected and measured by weight. The VAT from DIO male mice weighed (average weight = 2.10 g) almost three times that of control male mice (average weight = 0.77 g) (Figure 3A). The weight of VAT from DIO female mice (average weight = 0.78 g) was about 2–3 times as heavy as that of the control female mice (average weight = 0.27 g) (Figure 3B). Consistent with previous publications,^35^ our data also suggest that male mice accumulate more fat tissue when on a Western diet.

### 3.4 MAIT cells in Western diet‐fed female mice are not reduced

**Figure 4:**
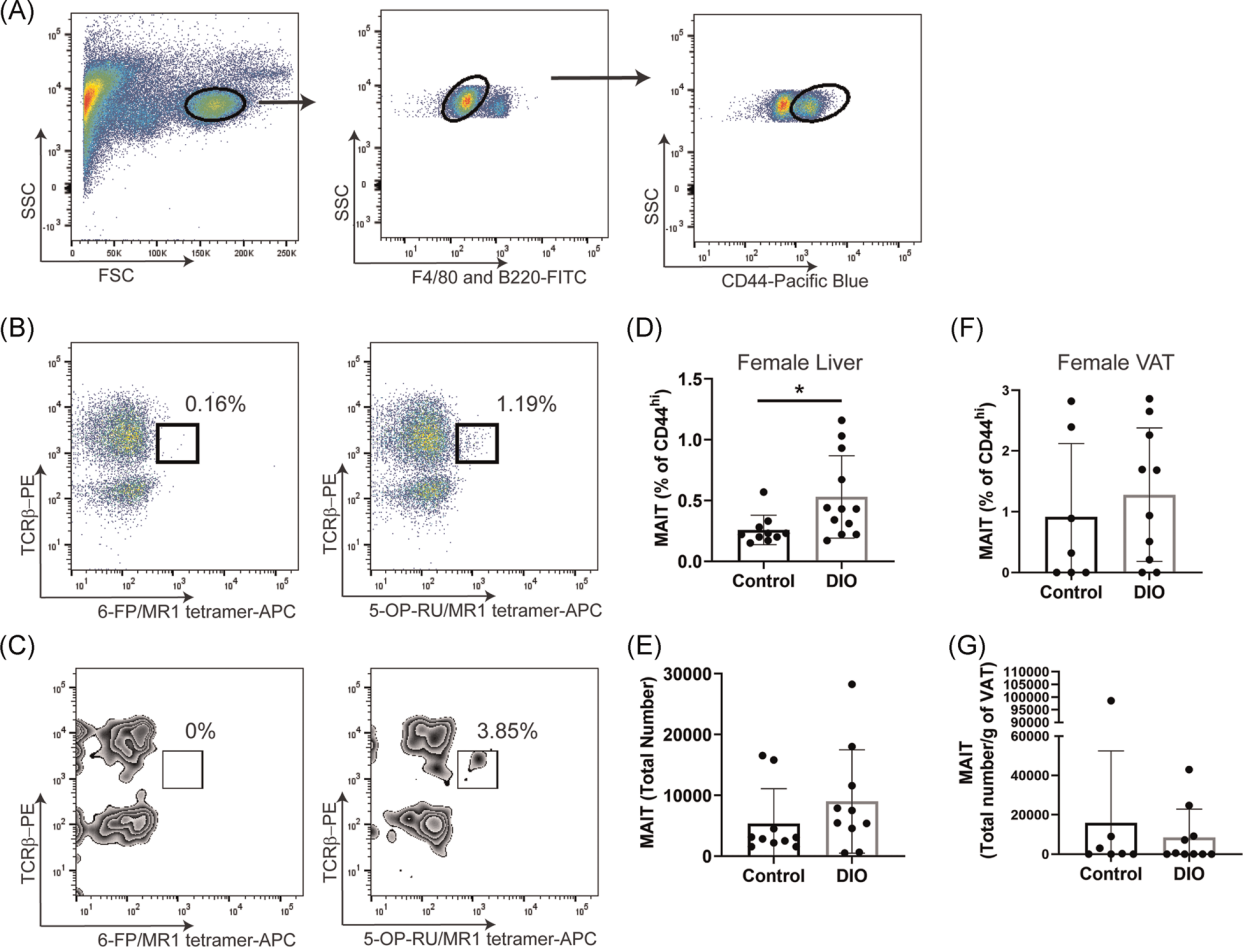
MAIT cells in female mice fed with a Western diet are at similar levels as those on a control diet. Female C57BL/6 mice were fed on either a Western DIO or a control diet for 10 weeks. Mouse liver mononuclear cells (MNCs) were isolated and stained with mAbs specific for F4/80, B220, CD44, TCRβ, and MR1 tetramers. The cells were analyzed by flow cytometry. The gating strategy for CD44^hi^ cells is shown in (A). Liver (B) and adipose (C) MAIT cells are shown as TCRβ^+^/5‐OP‐RU/MR1 tetramer^+^. A 6‐FP‐loaded MR1 tetramer was used as a control. Frequencies of liver (D) and VAT (F) MAIT cells, as well as total liver MAIT cells (E) and total MAIT cells per gram of VAT tissue (G) in the female mice, are summarized. The data are shown as the mean ± *SD*. Each dot represents an individual animal. DIO, diet to induce obesity; VAT, visceral adipose tissue

Mononuclear cells were isolated from liver tissues in female mice fed with a Western or control diet. These cells were then stained for MAIT cells. MAIT cells were identified as F4/80^−^B220^−^CD44^hi^TCRβ^+^5‐OP‐RU‐loaded MR1 tetramer^+^. A 6‐FP‐loaded MR1 tetramer was included as a negative control (Figure 4A,B). We found a wide range of MAIT cells in the liver and adipose tissues of female mice, whether they were on a Western diet or not. There was no difference in the percentage of MAIT cells between the Western diet and control groups (Figures 4D,F). Total MAIT cell numbers were also similar comparing Western diet‐fed female mice to those on a control diet (Figures 4E,G). Therefore, our data indicate that female mice on a Western diet do not have reduced MAIT cells.

### 3.5 Reduced MAIT cells in male mice with DIO

**Figure 5:**
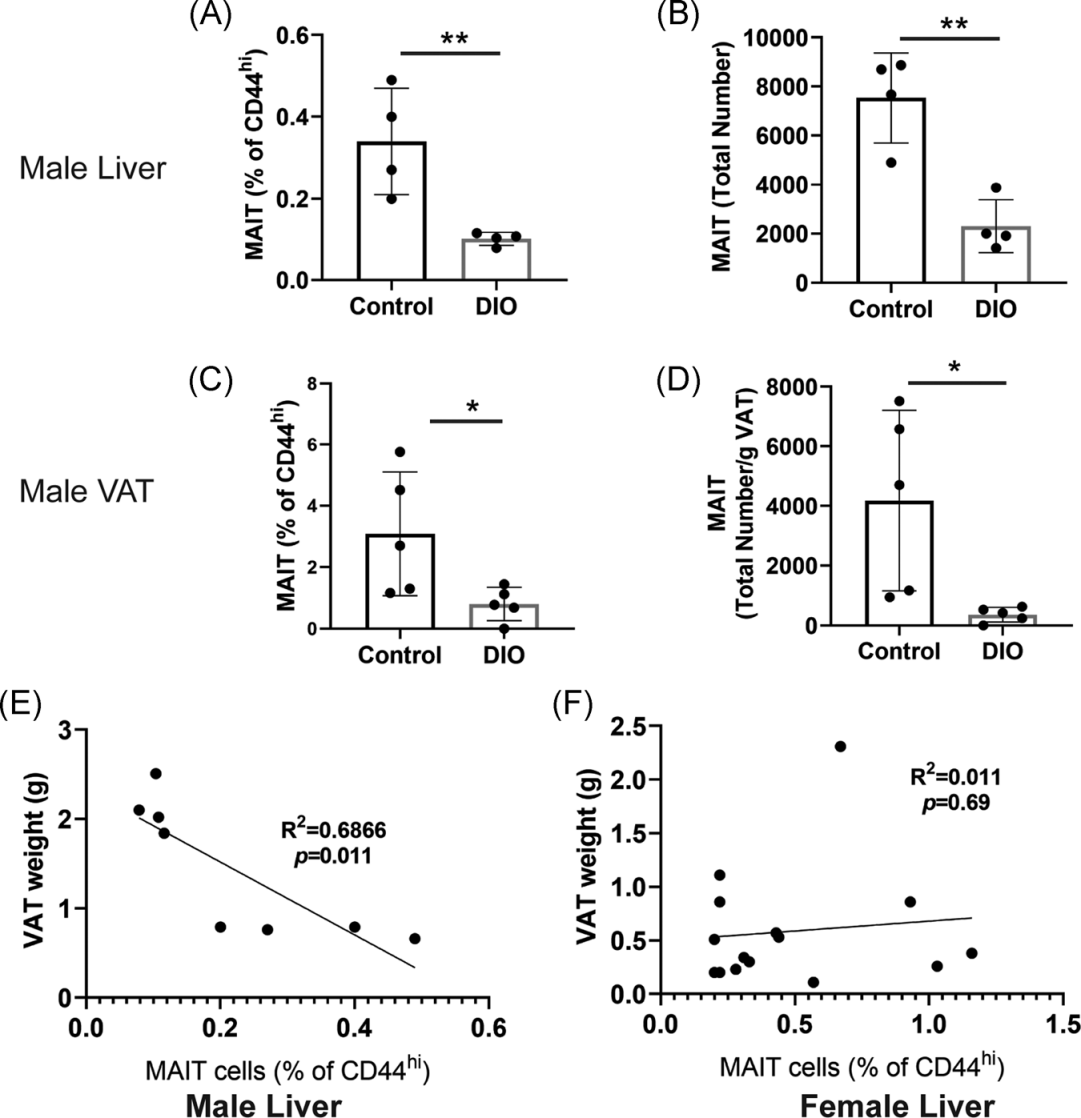
Reduced MAIT cells in male mice on a Western diet. Male C57BL/6 mice were fed on either a Western DIO or a control diet for 10 weeks. Mouse liver MNCs were isolated from male mice. The cells were stained as shown in Figure 4. Frequencies of MAIT cells as a percentage of CD44^hi^ cells (A, C) and total numbersof liver MAIT cells (B) and total numbers of MAIT cells per gram of VAT (D) are shown and summarized. The correlation between the frequencies of liver MAIT cells and VAT weights in male (E) and female (F) mice are shown. The data are presented as the mean ± *SD*. Each dot represents an individual animal. DIO, diet to induce obesity; MNC, mononuclear cell; VAT, visceral adipose tissue. *p < .05, **p < .01

MAIT cells from male mice on a Western diet were also analyzed. We found that male mice with DIO had significantly reduced MAIT cells in the liver (Figure 5A,B). The MAIT cell population in the adipose tissue was on average about ten times as that in the liver tissue (Figures 5A,C), and also significantly reduced percentage‐wise in Western diet‐fed male mice compared to those on the control diet (Figure 5C). Based on per gram of VAT tissue, total MAIT cells in the VAT of male mice on the Western diet was reduced as well, comparing to mice on the control diet (Figure 5D). Moreover and importantly, the percentages of MAIT cells in the livers of male (but not female) mice negatively correlated with VAT weights (Figure 5E,F). In conclusion, our data suggest that MAIT cells are enriched in VAT and male mice on a Western diet have fewer MAIT cells compared to those on a control diet.

## 4 DISCUSSION

In the current study, we found that there are fewer MAIT cells in mouse models of obesity and in obese patients, which is consistent with previous reports that only investigated obese patients.^20^, ^21^, ^30^ Because the expansion and maturation of MAIT cells depend on the microbial vitamin B matabolites in microbiota,^12^, ^14^, ^36^ it has been hypothesized that an altered microbiota in obesity reduces not only circulating MAIT cell numbers, but also their function. For example, MAIT cells from obese patients produce more of the proinflammatory cytokine IL‐17, but less of the anti‐inflammatory cytokine IL‐10, which may contribute to the increased insulin resistance observed in these patients.^20^, ^21^, ^30^ Reduced MAIT cell numbers and their impaired function in obesity may also exacerbate obesity‐associated inflammation. It is worthwhile to point out that we excluded obese patients with type 2 diabetes. Therefore, our study clearly shows that obesity itself can cause a MAIT cell reduction, confirming previous studies.^20^, ^21^

In the current study, we gated for mouse MAIT cells as a distinctive population from CD44^hi^ cells. CD44 is a cell adhesion receptor and widely expressed in different types of cells, including immune cells: NK, NKT, and memory T cells (T cells exposed to antigens prior) all express high CD44.^37^ Rahimpour et al. have shown that mouse MAIT cells from different tissues uniformly express high CD44.^38^ We also have a similar observation that majority (if not all) of MAIT cells express high CD44 (data not shown). We also found comparable frequencies of CD44^hi^ cells, TCRβ^+^ cells, as well TCRβ^+^ cells in CD44^hi^ populations in the control and DIO mouse groups (data not shown). Therefore, it was proper to compare the frequencies of MAIT cells in CD44^hi^ cells in the control and DIO groups. It is also known that, similar to mouse NKT cells, mouse MAIT cells also express NK cell marker NK1.1. Mouse MAIT cells in the liver mostly express NK1.1,^39^ but not in lung.^38^ It is unknown whether adipose MAIT cells express NK1.1.

It has been widely reported that sex difference plays a role in immune cell function and the development of a variety of infectious diseases.^40^, ^41^, ^42^ For example, sex hormones directly regulate gene transcriptional profiles in T cells and alter the function of thymic stromal cells.^41^ One study showed that female mice exhibited a more robust innate immune response, resulting in less morbidity in a urinary tract infection.^42^ Moreover, Group 2 innate lymphoid cells in the uterus, which are different from those in the lung, are altered by an ovariectomy and estrogen administration.^40^ NKT cells, sharing many similarities to MAIT cells as previously discussed,^43^, ^44^, ^45^ are also more abundent in women than in men.^46^, ^47^ Between the ages of 15–50, women have more MAIT cells compared to age‐matched men.^48^ In the current study, we also observed a trend of more MAIT cells in female subjects for the group aged 19–50 years old (data not shown). Furthermore, we found circulating MAIT cells in male obese patients were negatively impacted by obesity; this was not the case in female obese patients. Consistently, male (but not female) mice fed on a Western diet had reduced MAIT cells compared to those on a control diet. One caveat in our study is that the mean age of male healthy donors is much younger than that of male obese patients (Table 1, 42 vs. 51 years old). It has been shown that MAIT cells decreases as people age, especially after they turn 80 years old.^49^, ^50^ However, our data suggest that MAIT cells inversely correlate with age in female but not male subjects (Figures 1G,J). The age difference between the male obese and healthy donor groups in the current study should contribute very little to the difference of MAIT cell levels in these two groups. Therefore, we conclude that obesity reduces MAIT cells, especially in male subjects. In female subjects, the MAIT cell levels are not influenced by obesity, but rather by age. We speculate that certain sex hormones in female subjects can raise MAIT cell levels and MAIT cell levels may flucturate during menstrual cycles. After menopause, MAIT cell levels in female subjects may quickly decrease together with the reduction of sex hormones. Future research should focus on how sex hormones regulate MAIT cell development, distribution and function.

One caveat of the current study is that the sample size for obese patients and healthy subjects was quite small. The conclusions drawn from a study with sample size could have been largely influenced by outliers. For example, in Figure 1J, The conclusion that MAIT cell population does not inversely correlated with age in male could be due to one 53 years old man with 12% of MAIT cells, as an outlier. We also analyzed the age group of 19–50 years old to determine the association of MAIT cell frequencies with BMI in men and women. It was still evident that MAIT cell frequencies in men (but not women) are inversely associated with BMI aged 19–50 years old (data not shown).

Currently, flow cytometry is the typical method used to measure the frequency of MAIT cells. But this method is time‐consuming and requires expensive antibodies. We were intended to develop a more cost‐effective and faster method to quantify MAIT cells in the periphery. Real‐time PCR is the gold standard for gene expression quantification and it is also adaptable to high‐throughput assays.^22^ We initially validated the method of using qPCR to measure MAIT TCR Vα7.2 (*TRAV1.2*) expression. Our work suggests that it is possible to utilize qPCR to measure the levels of MAIT cells as shown in Figure 2; However, the data from the study cohort did not show any differences between obese and healthy groups. The primers and probe set we designed are for measurement of gene expression of Vα7.2, not Vα7.2^+^CD161^+^MAIT cells. The flow cytometry data suggest that the total number of Vα7.2^+^ cells is the same in the obese group as in healthy donors (Figure 1). This may partially explain why no difference in *TRAV1.2* expression was observed between the obese group and healthy donors (Figure 2A). The frequency of MAIT cells measured by flow cytometry also does not positively correlate with the values of *TRAV1.2* (Figure 2B); the same is true of the frequency of total Vα7.2^+^ cells (Figure 2C). These results therefore suggest that protein expression of Vα7.2 may not align with *TRAV1.2* messenger RNA expression. Lantz's group has reported that the Vα7.2‐Jα33 rearrangement is detectable in MAIT cells by polyclonal sequencing and qPCR, but not in Vα7.2^+^CD161^−^ cells.^11^ A primer and probe set for the Vα7.2‐Jα33 rearrangement, rather than only TCR Vα7.2 alone, are probably better for detecting MAIT cells by qPCR. Nonetheless, our work suggests that qPCR by itself is not ideal in terms of distinguishing small numerical differences in circulating human MAIT cells.

In conclusion, we found fewer human MAIT cells in samples from obese patients than from normal weight donors using flow cytometry. Although real‐time PCR has excellent potential in the measurement of MAIT cells, the qPCR method we used is not specific enough to detect the reduced human MAIT cells in obesity. Importantly, our work suggests that there is a sex discrepancy in the reduction of MAIT cells caused by obesity, that MAIT cell reduction is only observed in males (but not females) in both preclinical mouse models and human obese subjects. Future studies will focus on determining how obesity and sex hormones affect the function of MAIT cells.

## CONFLICT OF INTERESTS

The authors declare that there are no conflict of interests.

## AUTHOR CONTRIBUTIONS

Jianyun Liu, Randy R. Brutkiewicz, Kok Lim Kua, and Hongmei Nan designed the study. Jianyun Liu, Jose Casasnovas, and Kok Lim Kua performed the experiments and analyzed the data. Jianyun Liu, Kok Lim Kua, and Hongmei Nan wrote the manuscript.

## ETHICS STATEMENT

Blood samples from obese patients as well as healthy donors matched on age, sex, race/ethnicity, and fasting status were collected by the Indiana Biobank. This study was approved by the Institutional Review Board of Indiana University.

## Data Availability

The data that support the findings of this study are available from the corresponding author upon reasonable request.
